# Ethnic Differences in Body Composition and Obesity Related Risk Factors: Study in Chinese and White Males Living in China

**DOI:** 10.1371/journal.pone.0019835

**Published:** 2011-05-19

**Authors:** Dong Wang, Yanping Li, Simin Gharib Lee, Lei Wang, Jinhui Fan, Gong Zhang, Jiang Wu, Yong Ji, Songlin Li

**Affiliations:** 1 Civil Aviation Medicine Centre, Civil Aviation Administration of China, Chaoyang District, Beijing, China; 2 Chinese Centre for Disease Control and Prevention, Changping District, Beijing, China; 3 Department of Nutrition, Harvard School of Public Health, Boston, Massachusetts, United States of America; 4 Tsinghua University, Haidian District, Beijing, China; University Institute of Social and Preventive Medicine, Switzerland

## Abstract

The purpose of this cross-sectional observational study was to identify ethnic differences in body composition and obesity-related risk factors between Chinese and white males living in China. 115 Chinese and 114 white male pilots aged 28–63 years were recruited. Fasting body weight, height and blood pressure were measured following standard procedures. Whole-body and segmental body composition were measured using an 8-contact electrode bioimpedance analysis (BIA) system. Fasting serum glucose, fasting plasma total cholesterol (TC), high-density lipoprotein (HDL) cholesterol, and triglycerides (TG) were assessed using automatic biochemistry analyzer. After adjusting for age and body mass index (BMI), Chinese males had significantly higher percentage of body fat (PBF) both with respect to whole body (Chinese: 23.7%±0.2% vs. Whites: 22.4%±0.2%) and the trunk area (Chinese: 25.0%±0.3% vs. Whites: 23.2%±0.3%) compared to their white counterparts. At all BMIs, Chinese males had significantly higher fasting glucose levels (Chinese: 5.7±1.0 mmol/L vs. Whites: 5.2±1.0 mmol/L) but lower high-density lipoprotein levels (Chinese: 0.8±1.0 mmol/L vs. Whites: 1.0±1.0 mmol/L) than white males. In addition, a marginally significantly higher diastolic blood pressure was found among Chinese men than that among white men (Chinese: 80±1.0 mmHg vs. Whites: 77±1.0 mmHg). Chinese males had more body fat and a greater degree of central fat deposition pattern than that seen in white males in the present study. Furthermore, data on blood pressure, fasting glucose and blood lipids suggest that Chinese men may be more prone to obesity-related risk factors than white men.

## Introduction

Obesity is one of the greatest challenges facing global health experts today. Associated complications include increased risk of type 2 diabetes, cardiovascular diseases (CVD), and some cancers [1]. The growing burden of these complications is projected to result in substantial increases in health care expenditures and productivity loss [2].

In Asia, the world's most populous region, the epidemic pattern of obesity and its complications differs from that of the West, due to the considerable genetic and environmental heterogeneity in Asia. Some obesity comorbidities are similar or more prevalent in Asian countries than in Western countries, though Asians have lower rates of overweight and obesity than their Western counterparts [3]–[9]. For example, some studies suggest that type 2 diabetes and hypertension occur at a lower BMI and younger age for Asians than for Europeans [4]–[6]. In China, if overweight is defined according to the nation-wide China Health and Nutrition survey (CHNS) as BMI≥25 kg/m^2^ [10], less than 20% of Chinese adults under the age of 65 are overweight. In contrast, the prevalence of overweight among U.S. adults is approximately 65% according to the National Health and Nutrition Examination Survey III (NHANES III) [11]. Despite the difference in prevalence of obesity in China and the United States, the prevalence of obesity comorbidities in Chinese population is similar to those seen in the U.S. [12]–[14]. For example, the prevalence of diabetes in Chinese adults is 5.3% [14], which is comparable to the rate of 7.8% in Americans [13]. This trend suggests that the use of World Health Organization (WHO) cut-points may substantially underestimate the real burden of disease related to excess weight in non-Caucasians populations, especially in Asia [15]. Answering fundamental questions about obesity and its complications therefore requires ethnically comparative data. Gathering such comparative data, though, is challenging in Asian countries, especially in China, where such data are rare and conclusions are often derived from Asian immigrants living in Western countries [16]–[18].

Little is known about the apparent greater susceptibility of Asians to type 2 diabetes and CVD. However, differences in body fat distribution and body composition between Asian and Caucasian populations are thought to play some role in generating this disparity [6], [19]. Thus, more research is required to create ‘ethnic-specific’ BMI cut-off points that are predictive of obesity-related risk factors.

Comparative data on Chinese and Western populations are also limited and often lead to conflicting conclusions. When percentage of body fat (PBF) of Chinese is corrected by BMI, taking age and gender into account, some researchers found Chinese had a higher PBF for a given BMI level [16], [20]–[22], while other researchers had the opposite finding among Beijing Chinese [23], [24]. In another study, no difference in body fat was observed between a Dutch sample and Beijing Chinese sample [25].

This cross-sectional observational study provides a comparative analysis between Chinese and white male pilots focusing principally on body composition and obesity-related risk factors. We sought specifically to identify ethnic differences in (1) the relationship between body fat and body size, (2) fat distribution; and (3) associations between obesity-related risk factors and body size.

## Methods

### Study population

Subjects were 229 male pilots aged 28–63 years living in Shenzhen, Guangdong Province, China. The 114 Caucasian participants were from 15 European countries, 2 North American countries, 6 South American countries, Australia and South Africa. The inclusion criterion of these Caucasian subjects was that at least 3 of their 4 grandparents should be Caucasian. The subgroup of 115 Chinese were drawn from Caucasian subjects' colleagues in the same airlines. Informed written consent was obtained from all participants. Subjects with a history of cardiovascular, pulmonary, kidney, or malignant disease as well as those currently taking medications were excluded from the study. The study was approved by the Ethical Review Committee of the Civil Aviation Medicine Centre in China. We informed and explained our study methods, benefits and adverse reactions, objectives of this study, and definition of ethnicity to all subjects. Written consent was obtained from every subject before body composition measurement, physical examination, and blood sample collection. Subjects were instructed to fast and abstain from vigorous exercise for 12 hrs prior to physical examination.

### Bioimpedance measurement

An 8-contact electrode bioimpedance analysis (BIA) system (BC-418, Tanita Corp, Tokyo, Japan) was employed to measure body composition. Whole-body and segmental body composition was estimated using equations provided by the BIA manufacturer for all participants. The reliability and validity of this system has been proved both in Chinese and Caucasian populations [26], [27]. In addition, this system has been applied in a previous multi-ethnic study of body composition [28]. The procedure of the BIA measurement by this system has been described in detail in a previous study [26]. In this study, all subjects were measured once in the morning (between 8:30 and 10:30 a.m.) after the subjects had rested for 15 minutes by the same doctor (F. J. H.) over the course of 17 days (September 5, 2009–September 22, 2009) in a well-ventilated room with constant temperature and controlled relative humidity. BIA measurements were carried out at 50 kHz with a 0.8 mA sine wave constant current.

### Ascertainment of anthropometric indicators

The subject's body weight was measured to the nearest of 0.1 kg by a Weight–Tronix electronic scale (Scale Electronics Development, New York, USA). Height was measured without shoes to the nearest of 0.5 cm using a stadiometer (Holtain, Crosswell, Wales, UK). Body mass index (BMI) was calculated as weight (kg) divided by height (m) squared (kg/m^2^).

### Ascertainment of obesity related risk factors

All participants' blood pressures were measured by two physicians using standardized mercury sphygmomanometers. Two consecutive readings of blood pressure were taken in the right arm according to 1999 WHO/International Society of Hypertension guidelines on hypertension [29] with the participant in a seated position after 5 minutes of rest. The mean of the 2 measures was used for analysis. A venous blood sample (10 ml) was taken after a 12-hr fast before BIA measurement. Fasting glucose (FG), fasting plasma total cholesterol (TC), high-density lipoprotein (HDL) - cholesterol, and triglycerides (TG) were assayed by the same medical staff, using an automatic biochemistry analyzer (HITACHI 7600-020, Hitachi High-Technologies Corporation, Tokyo, Japan).

### Statistical analyses

The results are expressed as means and standard deviations. Any variables that were not normally distributed were logarithmically transformed prior to data analysis. Between-group differences in subject characteristics were tested by two-tailed T-test. Analysis of covariance (ANCOVA) was used to evaluate the associations between BMI and body composition, obesity-related risk factors and BMI, across different ethnic groups. Data were analyzed using SAS version 9.2 (SAS Institute Inc. Cary, NC.). Results with *P*<0.05 were considered significant.

## Results

Table 1 shows the characteristics and whole-body composition of all subjects. Chinese subjects were younger (*P*<0.01) and had lower body weight (*P*<0.01), height (*P*<0.01) and BMI (*P*<0.01) than Caucasian subjects. Caucasian subjects, on the other hand, had higher fat mass (FM) (*P*<0.01), fat-free mass (FFM) (*P*<0.01), and bioelectrical impendence (BI) (*P*<0.01). A two-tailed T-test shows that these differences between different ethnic groups are all significant except for PBF.

**Table 1:** Characteristics of subjects in different ethnic groups.

	Chinese		Whites		
	Mean	Std	Mean	Std	P
Age (years)	44	6	46	7	<0.01
Height (cm)	173.1	5.2	178.4	5.8	<0.01
Weight (kg)	76.5	8.5	84.3	10.5	<0.01
BMI (kg/m^2^)	25.5	2.3	26.5	3.0	<0.01
FM (kg)	17.9	4.0	19.6	5.6	<0.01
FM (%)	23.2	3.3	22.9	4.2	0.62
FFM (kg)	58.6	5.4	64.7	6.1	<0.01
BI (Ω)	563.6	56.7	528.1	51.6	<0.01

Continuous variables are given as mean values with their standard deviations.

BMI, body mass index, FM, fat mass, FFM, fat-free mass, BI, bioelectrical impendence.

Two-tailed T-test was applied to compare the differences between Chinese men and White men.

After adjusting for age and BMI, ANCOVA shows that the percentage of fat mass in whole body (*P*<0.01), trunk (*P*<0.01) and arm (*P*<0.01) were significantly higher in Chinese than that in Caucasians, with adjusted mean differences of 1.3%, 1.8% and 1.2%, respectively (Table 2).

**Table 2:** Body composition in different ethnic groups adjusted for age and BMI.

	Chinese		Whites		
	Mean	SEM	Mean	SEM	*P*
FM (kg)	18.7	0.2	18.9	0.2	0.52
FM (%)*	23.7	0.2	22.4	0.2	<0.01
TFM (%)*	25.0	0.3	23.2	0.3	<0.01
Arm FM (%)*	18.4	0.2	17.2	0.2	<0.01
Leg FM (%)	23.2	0.2	22.8	0.2	0.23

Continuous variables are given as mean values with their standard errors.

BMI, body mass index, FM, fat mass, FFM, fat-free mass, TFM, trunk FM.

Analysis of covariance (ANCOVA) adjusted for age (single years) and BMI (continuous).

*Mean values were significantly different between Chinese men and White men (*P*<0.05).

With respect to obesity-related risk factors, after adjusting for age and BMI, ANCOVA demonstrates that although Chinese men had higher DBP, FG, and TG, while Caucasian men had higher TC and HDL, only FG (*P*<0.01) and HDL (*P*<0.01) were statistically different between the groups. In addition, the adjusted mean value of DBP for Chinese men was 3 mmHg higher than that for white men (*P* = 0.06), representing a marginally significant difference (Table 3).

**Table 3:** Obesity-related risk factors in different ethnic groups adjusted for age and BMI.

	Chinese		Whites		
	Mean	SEM	Mean	SEM	*P*
SBP (mmHg)	119	1.0	119	1.0	0.95
DBP (mmHg)	80	1.0	77	1.0	0.06
FG (mmol/L)*	5.7	1.0	5.2	1.0	<0.01
TC (mmol/L)	5.1	1.0	5.4	1.0	0.15
TG (mmol/L)	1.6	1.1	1.5	1.1	0.61
HDL (mmol/L)*	0.8	1.0	1.0	1.0	<0.01

Continuous variables are given as mean values with their standard errors.

BMI, body mass index; SBP, systolic blood pressure; DBP, diastolic blood pressure; FG, fasting glucose; TC, total cholesterol; TG, triglyceride; HDL, high-density lipoprotein.

Analysis of covariance (ANCOVA) adjusted for age (single years) and BMI (continuous).

*Mean values were significantly different between Chinese men and white men (*P*<0.05).

## Discussion

This study is the first directly comparative study of body composition and obesity-related risk factors between Chinese and Caucasian male adults. For a given BMI, Chinese men had significantly higher PBF and more central fat deposition than their Caucasian counterparts. As for obesity-related risk factors, Chinese men had higher FG levels but lower HDL than Caucasians with the same BMI level.

Whole-body composition has been compared between Chinese and Caucasian populations in a limited number of previous studies [16], [19]–[25], [30], [31]. In this study, Chinese men had lower mean BMI than Caucasian men but higher PBF values, which is consistent with some of these studies' findings [16], [20]–[22], [31]. In addition, comparing the two nationally representative studies, NHANES III [32] and China National Nutrition and Health Survey 2002 (CNNHS 2002) [33], Chinese men held a relatively 15.0% lower mean value of BMI than that for American white men. While comparison results from two large-scale epidemiological studies, the Shanghai Diabetes Studies (SHDS) [34] and the NHANES III [35], show that the mean value of PBF for American men is relatively 7.4% higher than that for Chinese men. The relative difference of PBF between American and Chinese males is much less than the difference of BMI, implying that the PBF among American men should be lower than that of Chinese men with the same BMI level. Furthermore, among the multi-ethnic American adults from the NHANES III [36], PBF is generally lower for non-Hispanic whites than non-Hispanic blacks and then Mexican-Americans. Therefore, the difference for the mean value of PBF between American white men and Chinese men must be even lower. Consequently, the WHO classification of overweight (BMI≥25 kg/m^2^) and obesity (BMI≥30 kg/m^2^) [1] may underestimate the proportion of the Chinese males who are obese and the burden of obesity-related chronic diseases. In 2002, the Working Group on Obesity in China (WGOC) recommended BMI≥24 kg/m^2^ and BMI≥28 kg/m^2^ as the cut-off points for overweight and obesity respectively [37]. Though recognized internationally, the WGOC recommended cut-off points are based on studies that only investigated the relationship between BMI and obesity-related risk factors without considering the impact of body composition. The results of the present study add further support to changing the BMI thresholds for overweight and obesity for Chinese males.

Our results are consistent with the limited evidence for the propensity toward abdominal adiposity among Chinese [38]. There is also a clearly established stronger association between central obesity and risk for CVD and type 2 diabetes in Chinese populations [19], [28], [39]–[42]. Therefore, abdominal obesity (waist-hip ratio or waist circumference) and examination of visceral fat are more sensitive and specific measures for predicting diabetes or hypertension in Chinese.

In this study, for a given BMI, Chinese subjects showed significantly higher fasting glucose level but significantly lower HDL level than their Caucasian counterparts. Also, a marginally significantly higher DBP level was also observed among Chinese men. These results provide supporting evidence for the hypothesis that obesity-related risk factors may occur at a lower BMI for Asians than Europeans [3], [6], [31], [43], suggesting that Chinese populations may be at greater risk of developing type 2 diabetes and CVD. Furthermore, Pan et al. [32] and Colin et al. [7] directly compared obesity-related risk factors in Taiwan Chinese and U.S. Caucasians. They reported that increments of BMI corresponded to higher odds ratios (OR) in Chinese compared to U.S. Caucasian individuals for hypertension, hypercholesterolemia, hypertriglyceridemia, and diabetes. This trend among Chinese may be attributed to a strong genetic–environmental interaction, which has been intensified by rapid lifestyle changes in a growing Chinese economy [44]. Some evidence suggests exposure to under-nutrition in pregnancy followed by relative postnatal over-nutrition may also be a factor [45]. With China facing earlier onset and rapid increase in prevalence of type 2 diabetes and CVD [5], further research is necessary to discover the main cause for this study's findings and to prevent dangerous strain on the Chinese health care system.

This study has several limitations. First, although we recognized that the ethnic groupings we used in the current study did not take into account the potentially significant variation in genetic differences among Caucasian participants, we did not have sufficient data to produce reliable country-specific estimates among these populations. Furthermore, the findings in this study may not necessarily be generalizable to the population as a whole. Not only was our sample size relatively small, and entirely male, but the participants were also all pilots and thus probably younger and healthier than the general population. On the other hand, the associations that we studied (BMI-body composition, BMI-obesity-related risk factors) between different ethnic groups are all consistent with the findings of other studies, including those with more representative samples. In addition, all the pilots in this study did not receive any special physical training and medical care [46] because commercial airlines do not have such requirements, which would have influenced the results of this study.

In summary, this study demonstrates the marked differences in body composition, body fat distribution and obesity-related risk factors between Chinese and Caucasian males. These findings suggest that Chinese, even with ‘normal’ BMI, are more susceptible to obesity-related risk factors and make a case for lowering BMI standards for overweight and obesity (overweight: 24 kg/m^2^, obesity: 28 kg/m^2^) as recommended by China's Ministry of Health [34]. Considering the rapidly increasing rates of obesity and central adiposity in China, these epidemics threaten to overburden the health care system and create insurmountable public health challenges in China. The most cost-effective intervention for curbing these epidemics will likely require lifestyle modification [42] for the large, overweight and obese population in China as defined by the lower BMI levels recommended by the Ministry of Health. Moreover, because the WHO obesity and overweight cut-off points underestimate the susceptibility of Asian populations to obesity and overweight, these lifestyle modifications must also extend to Asian populations with a ‘normal’ BMI. China also needs to strengthen nation-wide early diagnosis capability, encourage effective management, and improve primary prevention measures to combat the growing disease burden due to this high-risk population. Meanwhile further research must be conducted to systematically monitor secular trends of obesity-related risk factors in China, characterize risk factors, and understand interactions between genetic and environmental risk factors. Finally, integrated strategy combining population-level preventive policies, early detection, and multidisciplinary care programs are needed to reduce the risk and associated complications in the general population and in high-risk individuals.
